# Severe Microbial Corrosion of L245 Transportation Pipeline Triggered by Wild Sulfate Reducing Bacteria in Shale Gas Produced Water

**DOI:** 10.3390/ma17174377

**Published:** 2024-09-04

**Authors:** Ming Sun, Xinhua Wang, Wei Cui, Hongfang Liu

**Affiliations:** 1 College of Mechanical & Energy Engineering, Beijing University of Technology, Beijing 100124, China; sm8014708@163.com (M.S.); luxinyuan66@126.com (X.W.); 2 China Special Equipment Inspection and Research Institute, Beijing 100029, China; 3 School of Chemistry and Chemical Engineering, Huazhong University of Science and Technology, Wuhan 430074, China

**Keywords:** shale gas pipeline, microbiologically influenced corrosion (MIC), sulfate-reducing bacteria (SRB), pitting corrosion, electrochemical measurements

## Abstract

The development of pitting corrosion on L245 carbon steel in a culture medium solution containing sulfate-reducing bacteria (SRB) was investigated. The results showed that the occurrence of corrosion in L245 carbon steel is closely linked to the evolution of biofilm and product film. As the test duration extended, overall corrosion was inhibited. Simultaneously, bacteria beneath the film layer promoted the generation and development of pitting corrosion, and the aggregation of bacteria (colonies) led to the aggregation of pitting corrosion.

## 1. Introduction

The oil and gas industry is closely related to energy security and socio-economic development, and an important part of it is fuel-gathering pipelines [1,2]. The internal structure and requirements of pipelines are favorable for the rapid proliferation of anaerobic microorganisms and biofilm formation, which ultimately cause microbiologically influenced corrosion (MIC) [3]. Sulfate-reducing bacteria (SRB) are the main typical microorganisms isolated from oil and gas sites and are also responsible for the localized corrosion of steel materials in oil and gas field gathering pipelines [4,5,6]. In addition, environmental factors such as CO_2_ and Cl^−^ in pipelines are coupled with SRB, resulting in a drastically reduced pipe life and corrosion perforation [7,8,9,10,11].

The primary threat of carbon steel from SRB biofilm lies in the rapid development of localized corrosion. Researchers have used filamentous electrode studies to investigate SRB corrosion, and the electrochemical effect in the presence of SRB promotes localized corrosion beneath deposits, resulting in a maximum pitting depth of approximately 7.7 times that of the control [12]. While biofilms may provide protection against overall corrosion [13], excessive extracellular polymeric substances (EPS) can also promote corrosion [14]. In pitting areas, cells aggregate at the bottom of the biofilm. Owing to the selective nature of anions, the accumulation of H^+^ ions, produced by microbial activity at the bottom of the biofilm, results in pitting [15].

MIC works are becoming more comprehensive with advances in biotechnology and characterization methods [16,17,18,19,20]. Two main types of MIC are now widely recognized: EET-MIC (extracellular electron transfer MIC) and M-MIC (metabolite MIC). For EET-MIC, in environments with a shortage of organic carbon and other electron donors, SRB biofilms acquire cathodic electrons from iron or other energetic metals by the EET pathway to generate energy, which causes MIC [21,22,23,24]. Organic carbon starvation would cause mature SRB biofilms to use iron as an electron source earlier, enhancing the pitting corrosion of metals by SRB [25,26,27,28,29]. Another M-MIC mechanism is the process of electrochemical corrosion with metal matrix using the metabolites of microorganisms as the corrosive medium. In general, the M-MIC pathway causes total corrosion of the metal rather than localized pitting. For example, H_2_S produced by SRB metabolism integrally thinned the Cu substrate without significant localized corrosion features on the corroded surface [30,31].

With shale gas extraction and hydraulic fracturing processes having advanced, the site of corrosion in transmission pipelines induced by SRB has increased significantly. SRB corrosion constitutes a significant factor in the deterioration of shale gas gathering pipelines. The mechanism of the corrosion behavior of SRB biofilm on the shale gas pipeline steel, L245, is not known. The development of pitting corrosion on L245 carbon steel in a culture medium solution containing sulfate-reducing bacteria (SRB) was investigated.

## 2. Material and Methods/Experimental

### 2.1. Biological Incubation

The production water was obtained from a shale gas gathering system. SRB was isolated and cultured using the following components of the culture medium, with each component per 1 L of deionized water: 0.5 g of K_2_HPO_4_·3H_2_O, 1.0 g of NH_4_Cl, 0.5 g of Na_2_SO_4_, 0.1 g of CaCl_2_, 2.0 g of MgSO_4_, 3.5 g of sodium lactate, 1.0 g of yeast extract, 0.3 g of Fe(NH_4_)_2_(SO_4_)_2_, and 0.1 g of vitamin C. The inoculation and cultivation methods followed the references [32]. The isolated bacteria were identified using the 16s rDNA technique.

We added 5 mL of the culture medium with SRB to 2 L of sterilized production water as the test solution. Before the corrosion test, the test container, polytetrafluoroethylene fixture, and deionized water were sterilized in an autoclave at a temperature of 121 °C for 20 min. The samples and chemicals underwent a 30 min sterilization process using ultraviolet light on a clean bench. The prepared test solution was deoxygenated by purging with 99.99% nitrogen gas for 24 h. The experiment periods were 1 day, 3 days, 7 days, 10 days, 15 days, and 21 days. The test temperature was maintained at 35 °C, and 5 mL of the culture medium with SRB was added to each group before the test commenced. The sterile test conducted over the same time period served as the control group.

### 2.2. Sample Preparation

L245 carbon steel was used for the experiments. The steel substrate has the following elemental composition: C (0.203%), Si (0.255%), Mn (0.397%), P (0.0189%), and S (0.0139%), with Fe constituting the balance. The test samples included three sizes: corrosion weight loss samples with dimensions of 60 mm × 20 mm × 3 mm for weight loss testing, with three parallel samples per group; morphology observation samples with dimensions of 20 mm × 20 mm × 3 mm, with two samples per group for the microscopic morphology observation and cross-sectional observation; electrochemical samples with dimensions of 10 mm × 10 mm × 3 mm, with three samples per group for open circuit potential and electrochemical impedance testing. For the electrochemical samples, only the top surface was exposed to the solution, and the other surfaces were painted with Teflon coating. The samples underwent pre-treatment, which involved grinding, polishing with #800 sandpaper, degreasing with acetone, drying with cold air, and subsequently with ultraviolet light for 30 min on a sanitized workbench.

### 2.3. Cell Counts and Corrosion Rate Measurement

The planktonic cell growth curves were measured using the most possible number (MPN) method on days 0, 1, 3, 7, 10, 15, and 21 from the solution. The 1 mL sample of target cells was extracted and injected into the SRB test vials purchased from Haitun. After inoculation, the test bottles were incubated in a bacterial incubator at 35 °C for one week, and the color of the test bottles was observed. All MPNs were repeated in at least three sets of ten vials each.

After the experiments, the corrosion weight loss samples were removed, rinsed with alcohol, dried with cold air, and then treated with Clack’s solution to remove the corrosion products. The corrosion rate was determined by weighing the samples. The weight loss data were used to calculate the corrosion rate:

(1) $$Corrosion\;Rate\;(mm/year)=\frac{8.76\;\times \;10^{4}\;\times \;\Delta m}{\rho \mathrm{St}}$$

where Corrosion Rate is the average corrosion rate, Δm (g) is the weight loss of L245 steel, S (cm^2^) is the surface area of that exposed, ρ (g/cm^3^) is the density of metal, and t (h) is the time of immersion in the medium.

Three-dimensional measurements were conducted using a three-dimensional stereo microscopy system (CLSM, VHX-10000, Keyence, Japan) with the surface-film-layer-removed samples.

### 2.4. Corrosion Morphology and Surface Composition

Following the experiments, the samples were taken out, rinsed with alcohol, dried with cold air, and observed under a scanning electron microscope (SEM, s-3400N, Hitachi, Tokyo, Japan) for the microscopic morphology, along with energy-dispersive X-ray spectroscopy (EDS) analysis. The cross-sectional samples underwent cold-embedding, were polished with #800 sandpaper, rinsed with alcohol, dried with cold air, and subsequently gold-sputtered on their surfaces. This allowed for the observation of the distribution of the film layer, and an analysis of element mapping was conducted.

The samples with the surface film layer removed were observed under a scanning electron microscope to study the pitting morphology and distribution. After 21 days of immersion, X-ray photoelectron spectroscopy (XPS, ESCALAB 250Xi, Thermo Fisher Scientific, Waltham, MA, USA) was performed to check the components of the corrosion products and passive layers.

### 2.5. Electrochemical Testing

Electrochemical measurements were conducted to assess the surface state changes of the samples using an electrochemical workstation. Electrochemical tests with a three-electrode system and equipped with an electrochemical workstation (CHI 660E, CH Instruments, Shanghai, China) were carried out in a 500 mL glass bottle containing 400 mL of culture medium and 1 mL of SRB. The counter electrode was a 20 × 20 mm^2^ platinum electrode. The reference electrode was a saturated calomel electrode (SCE). The working electrodes were three epoxy-sealed L245s.

Open circuit potential (OCP) and electrochemical impedance spectroscopy (EIS) were carried out. In the EIS test, the amplitude of the sine wave excitation signal was 20 mV, and the sweep frequency range was from 0.01 Hz to 10^5^ Hz. Tafel curves were scanned from −0.5 V (vs. OCP) to the breakdown potential with a scanning rate of 0.5 mV·s^−1^. All tests were performed at 40 °C.

## 3. Results

### 3.1. Cell Identification and Culture Counting

**Figure 1:**
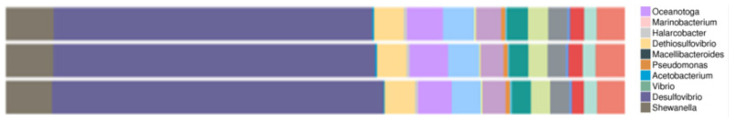
Strip chart of bacterial identification.

The bacterial identification results in Figure 1 mainly revealed the presence of *Desulfovibrio* sp., classified as a type of mesophilic bacterium, as seen in the strip chart below. These cells are curve- or rod-shaped, occurring either singly or in pairs. Simple organic compounds such as lactate can serve as the electron donor and carbon source for these anaerobic bacteria with respiratory and fermentative metabolism [33,34].

**Figure 2:**
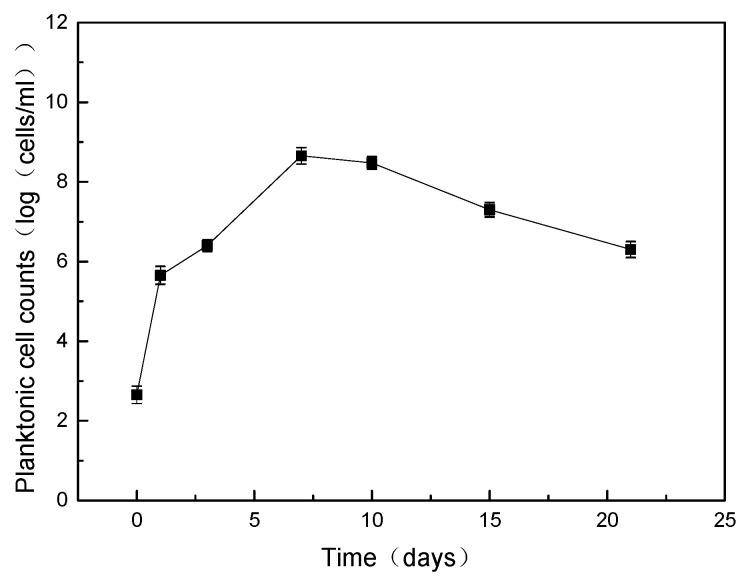
SRB concentration after the experiments.

After 21 days of incubation, the pH value of the culture medium was 7.6, which ruled out the possibility of acid corrosion. The results of SRB cell counting are shown in Figure 2. The initial bacterial concentration was about 450 cells/mL, and the SRB cells showed exponential growth from day 1 to day 7, reaching a peak of 4.5 × 10^8^ cells/mL on day 7. Compared with day 7, the concentration of SRB cells on day 10 was slightly decreased. From day 10 to day 21, due to the lack of nutrients, the bacterial concentration further decreased to 2 × 10^6^ cells/mL on day 21.

The number of bacteria associated with the steel surface was 7.15 × 10^7^ cells/mL on day 7 and decreased to 5.05 × 10^7^ cells/mL on day 21. 

### 3.2. Corrosion Rate Calculation

**Figure 3:**
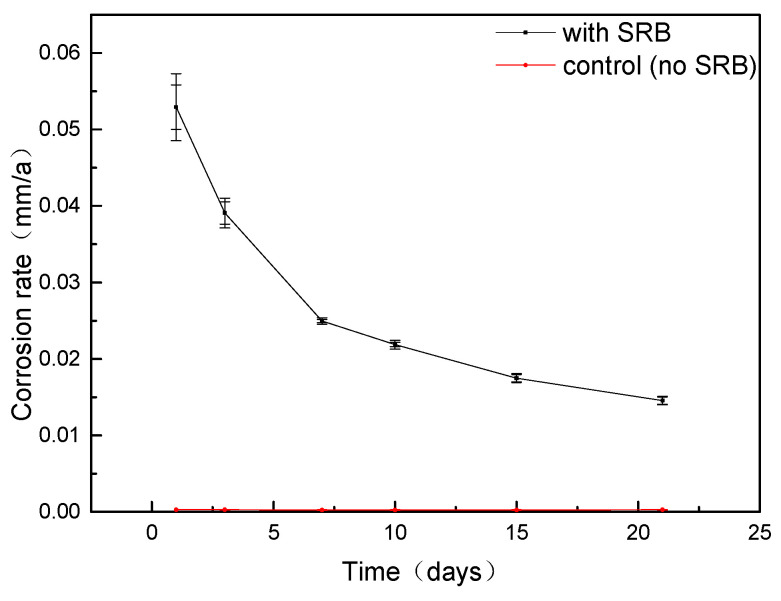
General corrosion rate of L245 incubating in SRB medium.

The general corrosion rate of L245 was calculated using the weight loss method, and the results are depicted in Figure 3. The average corrosion rate exhibited a decreasing trend with time. The corrosion rate exhibited higher values at 1 day at 0.0529 mm/year and 3 days at 0.0391 mm/year, followed by a significant decrease at 7 days at 0.0250 mm/year and 10 days at 0.0219 mm/year. The corrosion rate on the tenth day decreased by 44% compared to the third day, and the lowest CR value was obtained at 21 days, which was 0.0146 mm/year. These values are all higher when compared to the control.

### 3.3. Incubation Surface and Cross-Section Observations

**Figure 4:**
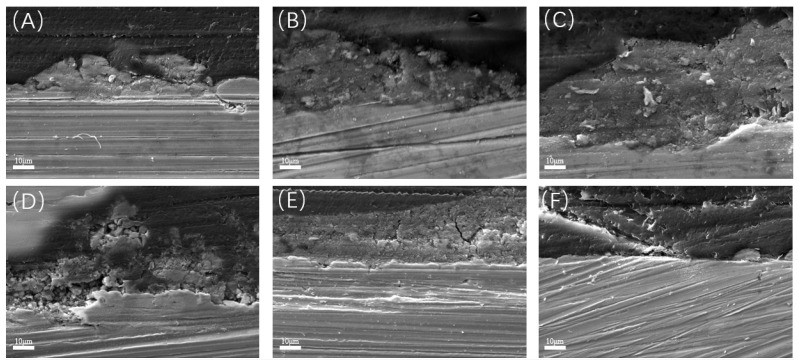
Cross-sectional morphology observation over time: (**A**) day 1; (**B**) day 3; (**C**) day 7; (**D**) day 10; (**E**) day 15; (**F**) day 21.

**Figure 5:**
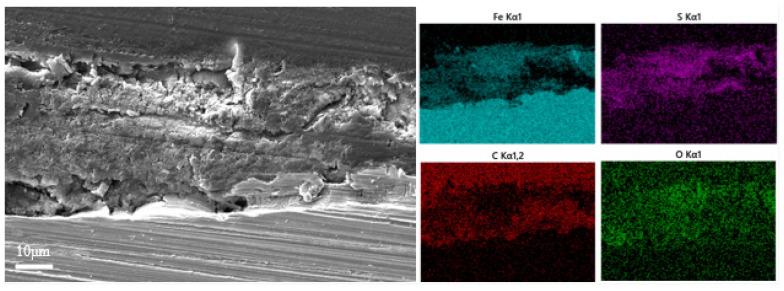
Cross-sectional morphology observation and element mapping results of the sample immersed for 7 days.

**Figure 6:**
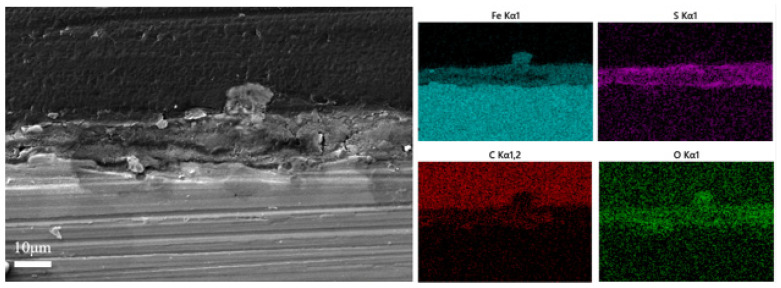
Cross-sectional morphology observation and element mapping results of the sample immersed for 21 days.

Figure 4 exhibits the variations in the thickness of the surface film layer of L245 steel. The film thickness increased to about 30 μm for the first 7 days of incubation and then thinned down, attributed to the shedding of biofilm. Cross-sectional observation and elemental mapping of the samples immersed for 7 days, in Figure 5, revealed that the main elements in the film layer were Fe, S, C, and O. The main components of corrosion products were Fe and S [35], which implies the main corrosion product is probably FeS and was found in higher concentrations in the outer layer, while C and O were the major elements in the extracellular polymeric substance (EPS) of the biofilms [36], which the proteins and other organic metabolites were the main components and were mainly found in the inner layer. However, the overall structure of the film layer is a mixture with no distinct boundaries. Further cross-sectional observations and elemental mapping were carried out on the coupons immersed for 21 days (Figure 6). The main components of the film layer were still Fe, S, C, and O. The thickness decreased from about 30 µm at day 7 to 10 µm at day 21.

**Figure 7:**
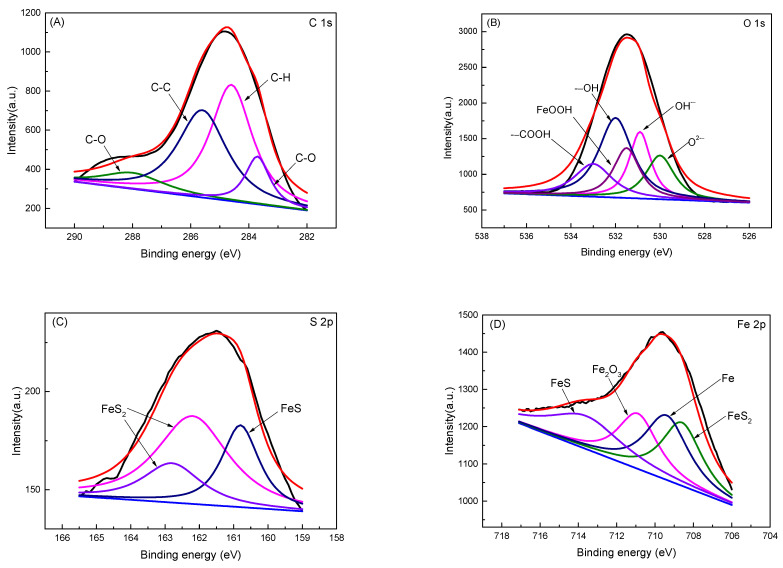
XPS spectra of biofilm and corrosion products after incubating for 21 days at 35 °C in the medium: (**A**) C 1s; (**B**) O 1s; (**C**) S 2p; (**D**) Fe 2p. Black lines are the original spectral lines and red lines are the fitted lines.

Figure 7 shows the high-resolution XPS spectra of C 1s, O 1s, S 2p, and Fe 2p for samples of the biofilm and corrosion products after incubating for 21 days in the SRB medium. In the C 1s spectra, peaks at 288.00 eV/283.70 eV, 285.60 eV, 284.80 eV, and 283.70 eV corresponded to C-O, C-C, and C-H, respectively, which were important components of the SRB biofilm. In the O 1s spectra, peaks at 533.20 eV, 532.00 eV, 531.40 eV, 531.20 eV, and 530.10 eV corresponded to -COOH, -OH, FeOOH, OH^−^, and O^2−^, respectively. In the S 2p spectra, peaks at 162.80 eV/162.20 eV and 160.80 eV corresponded to FeS_2_ and FeS, respectively, which confirmed the presence of Fe and S compounds in the mapping spectra (Figure 5 and Figure 6). In the Fe 2p spectra, peaks at 713.60 eV, 711.00 eV, 709.40 eV, and 708.60 eV corresponded to FeS, Fe_2_O_3_, Fe, and FeS_2_, respectively. Chemical bonds of -COOH and -OH are components of nucleic acids and cell walls [15]. The main composites of the corrosion products are iron oxides (FeOOH, Fe_2_O_3_) and iron sulfide (FeS, FeS_2_).

### 3.4. Pitting Observation and Measurement

**Figure 8:**
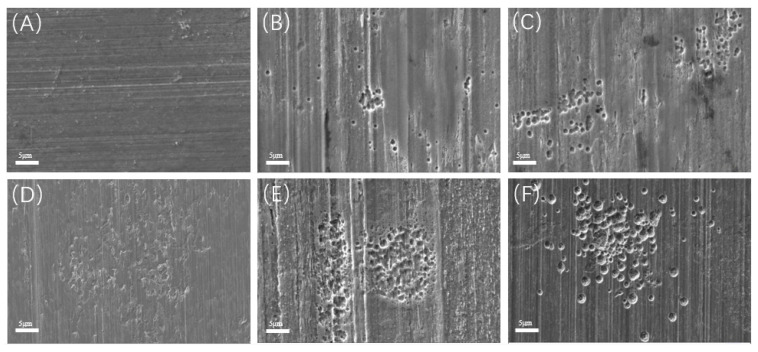
SEM images of the acid-washed sample in different test periods: (**A**) day 1; (**B**) day 3; (**C**) day 7; (**D**) day 10; (**E**) day 15; (**F**) day 21.

**Figure 9:**
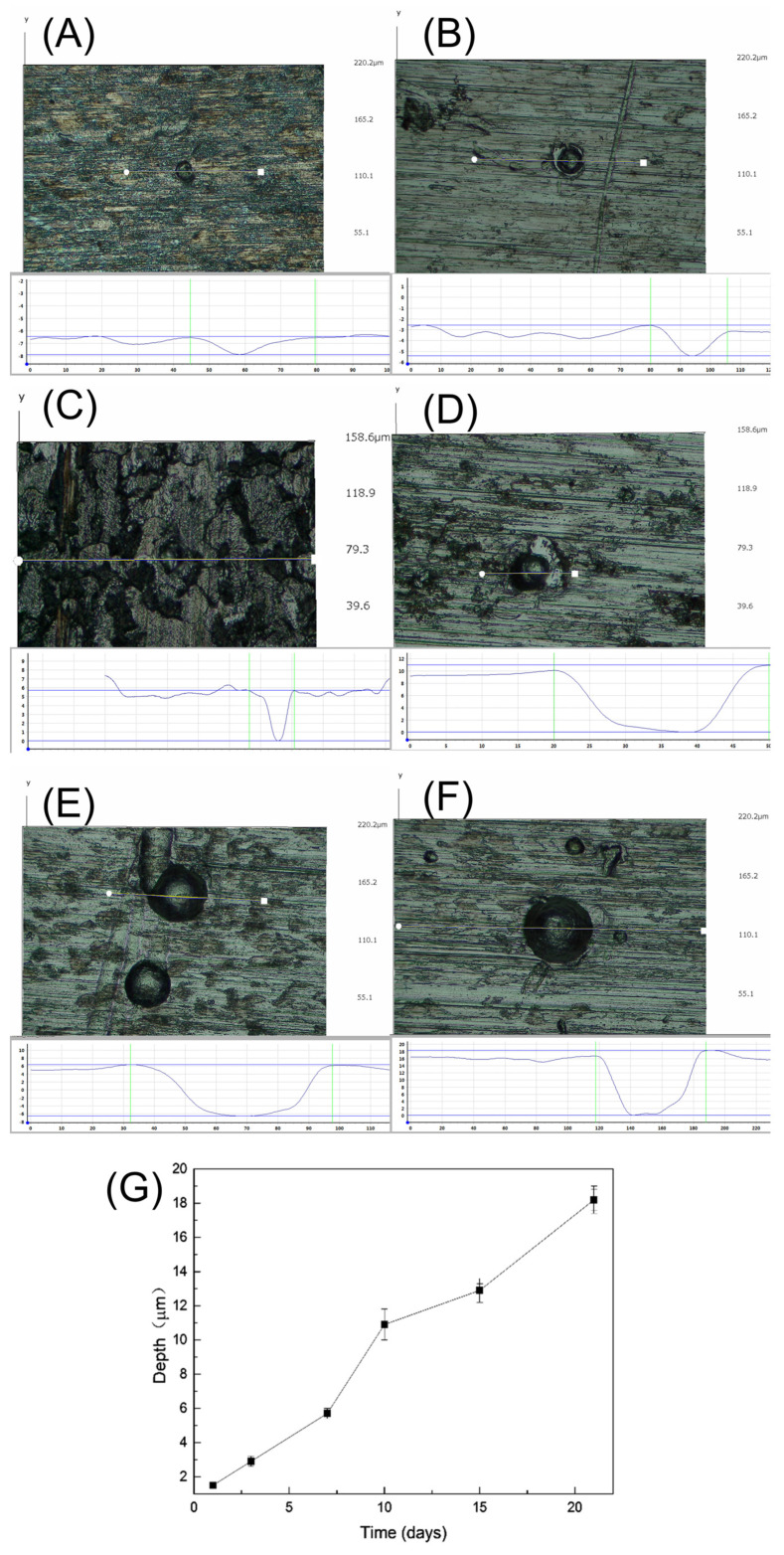
Pitting depths measured with a three−dimensional stereomicroscope system: (**A**) day 1; (**B**) day 3; (**C**) day 7; (**D**) day 10; (**E**) day 15; (**F**) day 21; (**G**) pitting depths.

After the removal of biofilm and corrosion products, the corrosion profiles with different incubation times were observed on the surface of the metal coupons. As shown in Figure 8A, there were no obvious pits at the initial stage of corrosion. After 3 days of immersion (Figure 8B), very few pits appeared, which were small, shallow, and irregular in shape. After 7 days of immersion, as shown in Figure 8C, small round holes began to appear and tended to cluster. With the increase in immersion time, the pitting was gradually denser and deeper, as shown in Figure 8D–F. Typical pitting depths at different time periods were tested and compared using a 3D stereo microscope system, as shown in Figure 9. The maximum pitting depth increased from 1.5 μm to 5.7 μm for the immersion times from 1 to 7 days. After day 7, the pitting depth increased rapidly and reached a maximum pitting depth of 18.2 μm at day 21.

### 3.5. Electrochemical Test Results

**Figure 10:**
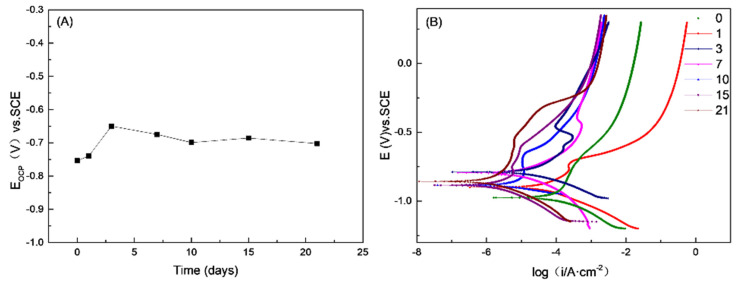
OCP curves vs. time during 21 days of incubation (**A**) and potentiodynamic polarization curves at the end of immersion (**B**) in SRB medium.

With an extended testing time, the biofilm and product film layers on the sample surface undergo changes and have an impact on the electrochemical behavior of carbon steel. After being immersed for 0–21 days, an open circuit potential (OCP) curve was conducted, as shown in Figure 10. From 0 to 1 day, the OCP shifted from −0.7035 V to −0.6898 V vs. SCE, and from 1 to 3 days, it further shifted to −0.5997 V vs. SCE. From 0 to 3 days, the OCP remained in the positive offset stage, indicating anodic polarization and rapid corrosion development on the sample surface. Starting from day 7, the open circuit potential no longer exhibited a positive shift. Instead, it exhibited a slight negative shift from day 7 to day 10 and then remained stable from day 10 to day 21. This stability suggests the formation of a relatively dense film layer on the sample surface.

**Figure 11:**
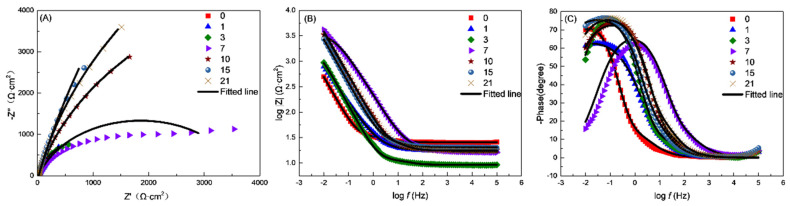
Nyquist (**A**) and Bode (**B**,**C**) plots of L245 steel immersed in SRB medium for 1, 3, 7, 10, 15, and 21 days.

**Figure 12:**
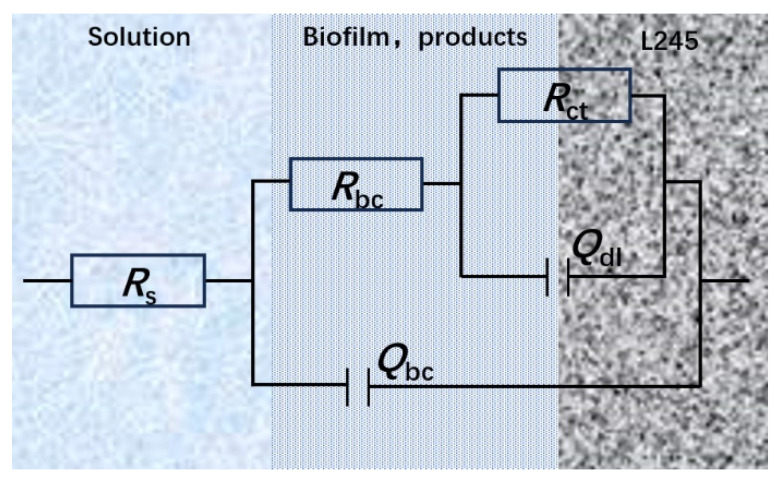
Equivalent circuits for EIS fitting.

Figure 11 displays the EIS plot with fitting lines, and the fitting values and equivalent circuits are presented in Table 1 and Figure 12. A two-time constant circuit was used to simulate the results. *R*_s_, *R*_bc_, and *R*_ct_ stand for the solution resistance, the resistance of the biofilm and corrosion product layer, and the charge transfer resistance, respectively. *Q*_bc_ and *Q*_dl_ stand for the capacitance of the biofilm and corrosion product film and the double-layer capacitance, respectively.

**Figure 13:**
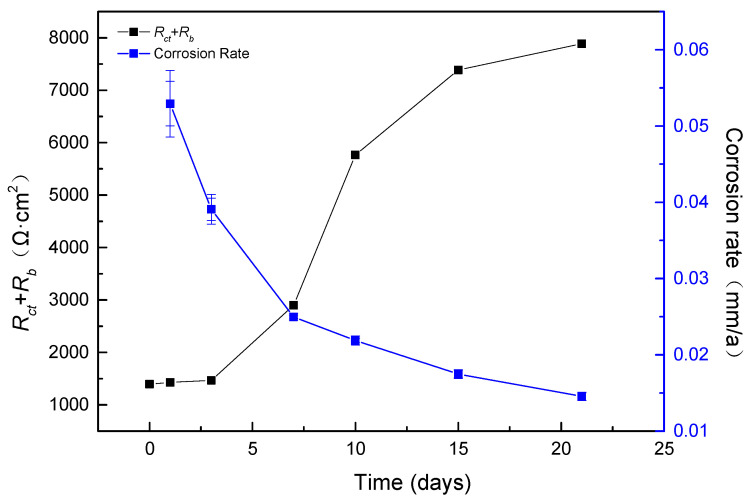
Time-dependent curves of EIS parameters *R*_ct_ + *R*_bc_.

As shown in Figure 11 and Table 1, over the course of the 21-day experiment, *R*_s_ remained consistently stable. *R*_ct_ was lower from day 0 to day 3, indicating a higher corrosion rate during this period. It significantly increased from day 7 to day 21, suggesting a lower corrosion rate during this stage. The combined parameter *R*_ct_ + *R*_bc_ exhibited a similar pattern. *R*_ct_ + *R*_bc_ is negatively correlated with the corrosion rate, as shown in Figure 13.

The rise in *R*_ct_ + *R*_bc_ indicated a hindered electron transfer from the electrolyte solution to the electronic double layer. When a mature biofilm and corrosion product formed on the carbon steel sample’s surface, a finite diffusion layer emerged between the bulk solution and the electronic double layer. This layer consisted of the biofilm and corrosion product. Therefore, taking into account this transfer barrier, the collection of electrons from the carbon steel sample proved to be more efficient than from other electron donors in the bulk solution [15].

## 4. Discussion

Pitting corrosion poses a greater threat to the integrity of metals than uniform corrosion [37]. MIC pitting is often explained as the breakdown of protective films (such as FeS or passivation films in stainless steel) [23,38,39,40]. However, in this study, no correlation was found between film damage and pitting. Therefore, MIC pitting must be explained by mechanisms rather than damage to FeS films or less passivated films.

**Figure 14:**
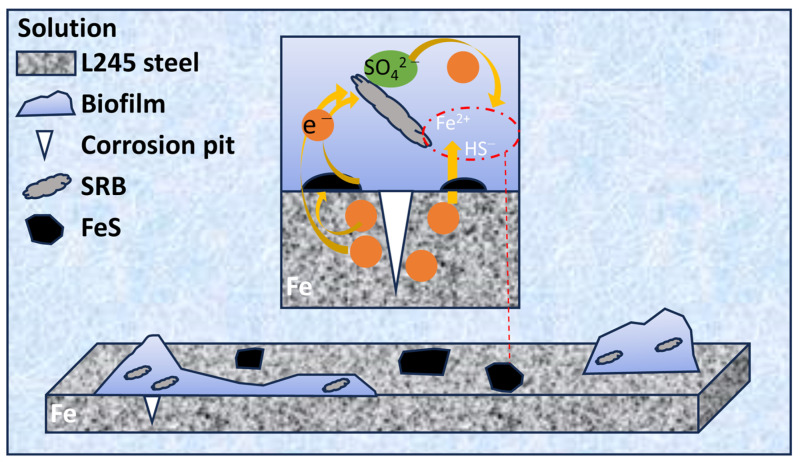
Corrosion mechanism diagrams of L245 steel in biotic solution.

When the SRB culture seed was added, the formation of biofilm and the corrosion product layer was not complete, as shown in Figure 14. As the growth of biofilm and the formation of corrosion products occurred, the *R*_bc_ + *R*_ct_ increased. A rising *R*_bc_ + *R*_ct_ value implies that the electron transfer from the medium solution to the electron double layer was hindered, and a limited diffusion layer exists between the bulk solution and the electron double layer, which is the biofilm and corrosion product. The presence of biofilms and corrosion products forms a protective barrier between the substrate and the external corrosive environment, consequently reducing the corrosion rate. Nevertheless, this film layer also impedes direct contact between the SRB and external carbon sources. Thus, harvesting electrons from iron samples is more efficient than from other electron donors in the medium solution, considering the transfer barrier. As a result, SRB resorts to extracting electrons directly from the metal to sustain their physiological activities, ultimately resulting in pitting. It is logical for SRB cells situated at the bottom of the film layer to absorb electrons from the carbon steel sample, as it is more accessible for them compared to absorbing electrons from the medium solution. The research indicates that SRB biofilms and corrosion products exhibit an anion-selective characteristic, causing cations like H^+^ to be sequestered beneath them. This phenomenon encourages acidification beneath the biofilms, consequently contributing to pitting [15].

**Figure 15:**
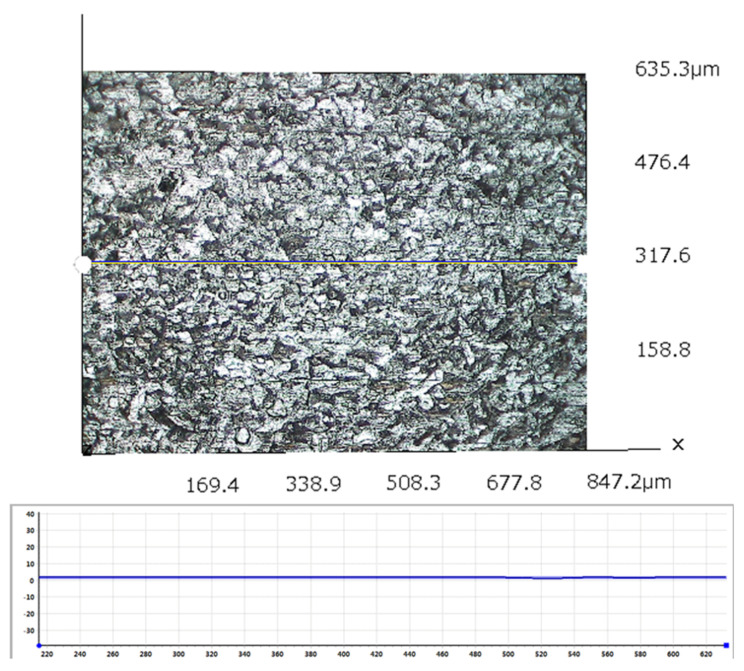
Pitting depths measured with a three-dimensional stereomicroscope system after 21 days without SRB.

As shown in Figure 15, the pitting in the bacterial-containing test groups is much more severe than the control without SRB. In the bacterial-containing test groups, the film layer began to form from day 7 onward, and by day 21, cracks appeared in the film. During this period, the film layer remained intact, but upon its removal, pitting became evident on the substrate. With time, the area, depth, and quantity of pitting increased, indicating that the occurrence and progression of pitting were influenced by actions beneath the film layer. Pitting became widespread starting from day 7, suggesting that bacteria require some time to exert their effects on the substrate surface. Starting from day 7, pitting began to exhibit a clustering effect. Similar situations were observed on days 10, 15, and 21, as shown in Figure 8, which were associated with the growth of bacterial colonies.

## 5. Conclusions

Surface corrosion on carbon steel samples is intimately linked with the development of biofilms and corrosion product films. As the experiment advances, the film layer envelops the entire substrate surface, thickening in the process. This prompts a notable surge in film resistance and charge transfer resistance, effectively inhibiting the overall corrosion. Then, the membrane layer detached locally, and the rate of impedance increase and corrosion rate decrease slowed down.

While this inhibition of overall corrosion takes place, bacteria situated beneath the film layer are compelled to alter their growth mode, drawing electrons from Fe^0^. This catalyzes the initiation and progression of pitting.

While the film layer induces severe pitting, it also furnishes a shield against uniform corrosion in terms of weight loss.

## Figures and Tables

**Table 1:** EIS parameters of L245 coupons during the 21 days of incubation in the SRB medium.

	*R*_s_ (Ω·cm^2^)	*Q*_bc_ (Ω^−1^·cm^−2^·s^n^)	*R*_bc_ (Ω·cm^2^)	*Q*_dl_ (Ω^−1^·cm^−2^·s^n^)	*R*_ct_ (Ω·cm^2^)	*R*_bc_ + *R*_ct_ (Ω·cm^2^)
0	25.97	9.28 × 10^−3^	24.09	1.88 × 10^−2^	1372	1396.09
1	18.14	4.86 × 10^−3^	99.11	9.89 × 10^−3^	1331	1430.11
3	9.553	6.59 × 10^−3^	34.18	5.95 × 10^−3^	1432	1466.18
7	17.68	4.55 × 10^−4^	286	8.41 × 10^−4^	2612	2898
10	19.06	1.89 × 10^−3^	340.3	1.74 × 10^−3^	5427	5767.3
15	21.01	2.27 × 10^−3^	132.4	2.48 × 10^−3^	7255	7387.4
21	18.14	1.48 × 10^−3^	116.6	1.63 × 10^−3^	7772	7888.6

## Data Availability

Data will be made available on request.
